# Roles of HMGBs in Prognosis and Immunotherapy: A Pan-Cancer Analysis

**DOI:** 10.3389/fgene.2021.764245

**Published:** 2021-10-29

**Authors:** Tong Lin, Yingzhao Zhang, Zhimei Lin, Lisheng Peng

**Affiliations:** ^1^ The Fourth Clinical Medical School, Guangzhou University of Chinese Medicine, Shenzhen, China; ^2^ Department of Science and Education, Shenzhen Traditional Chinese Medicine Hospital, Shenzhen, China

**Keywords:** HMGB, pan-cancer, prognosis, immunotherapy, biomarker

## Abstract

**Background:** High mobility group box (HMGB) proteins are DNA chaperones involved in transcription, DNA repair, and genome stability. Extracellular HMGBs also act as cytokines to promote inflammatory and immune responses. Accumulating evidence has suggested that HMGBs are implicated in cancer pathogenesis; however, their prognostic and immunological values in pan-cancer are not completely clear.

**Methods:** Multiple tools were applied to analyze the expression, genetic alternations, and prognostic and clinicopathological relevance of HMGB in pan-cancer. Correlations between HMGB expression and tumor immune-infiltrating cells (TIICs), immune checkpoint (ICP) expression, microsatellite instability (MSI), and tumor mutational burden (TMB) in pan-cancer were investigated to uncover their interactions with the tumor immune microenvironment (TIME). Gene set enrichment analysis (GSEA) was conducted for correlated genes of HMGBs to expound potential mechanisms.

**Results:** HMGB expression was significantly elevated in various cancers. Both prognostic and clinicopathological significance was observed for *HMGB1* in ACC; *HMGB2* in ACC, LGG, LIHC, and SKCM; and *HMGB3* in ESCA. Prognostic values were also found for *HMGB2* in KIRP and MESO and *HMGB3* in BRCA, SARC, SKCM, OV, and LAML. The global alternation of HMGBs showed prognostic significance in ACC, KIRC, and UCEC. Furthermore, HMGBs were significantly correlated with TIIC infiltration, ICP expression, MSI, and TMB in various cancers, indicating their regulations on the TIME. Lastly, results of GSEA-illuminated genes positively correlated with HMGBs which were similarly chromosome components participating in DNA activity-associated events.

**Conclusion:** This study demonstrated that HMGBs might be promising predictive biomarkers for the prognosis and immunotherapeutic response, also immunotherapy targets of multiple cancers.

## Introduction

Immunotherapy has revolutionized the treatment landscape of patients with advanced cancers, especially immune checkpoint inhibitors (ICIs). Immune checkpoints (ICPs), such as programmed death protein 1 (PD-1) and its ligand (PD-L1), are negative modulatory signaling pathways for activation of T cells, which in turn facilitate immune tolerance and promote cancer. ICIs aim to unleash T cells from exhaustion and enhance anticancer immune activity. However, only 20% of patients derive the response to ICIs across all malignancies, which severely limits their clinical benefits (Rameshbabu et al., 2021). Therefore, seeking new immunotherapeutic targets and predictive biomarkers for immunotherapy efficacy for patient selection is a hot issue of the current research (Yi et al., 2018).

The high mobility group box (HMGB) protein family, consisting of HMGB1-4, includes non-histone chromatin components (Rapoport et al., 2020). HMGB1-3 share over 80% identical sequence and structure, comprising two DNA-binding domains and an acidic tail. However, HMGB4 lacks the acidic tail and is not ubiquitously expressed like HMGB1-3 (Taniguchi et al., 2018). This study focused on HMGB1-3. HMGBs are predominantly in the nucleus and act as DNA chaperones, thereby modulating chromosome stabilization, telomerase maintenance, replication, transcription, and DNA repair (Cheng et al., 2020). In the cytoplasmic or extracellular milieu, HMGBs act as chemokines or cytokines to evoke inflammatory and immune responses (Niu et al., 2020).

Accumulating evidence had hinted HMGBs’ participation in cancer pathogenesis. First, effective DNA damage repair is indispensable for cancer cells to maintain growth. Second, excessive extracellular HMGBs induce chronic inflammation, which is a hallmark of cancer (Mukherjee and Vasquez, 2020). The overexpression and prognostic relevance of HMGBs had been observed in various cancers, including prostate (Jung et al., 2021), liver (Zhang et al., 2014), cervix (Cheng et al., 2017; Li T. et al., 2020), breast (Fu et al., 2018), stomach (Cui et al., 2019), esophagus (Gao et al., 2015), and hematopoietic malignancies (Yuan et al., 2020). Given the roles of HMGBs in the regulation of inflammation and immunity, they appear to be candidate targets for cancer immunotherapy. However, HMGB1 is double-faced in cancers. HMGB1 can maintain genome stability and interact with tumor suppressor proteins, e.g., Rb, to prevent oncogenesis (Mandke and Vasquez, 2019). Besides, extracellular HMGB1 can stimulate anticancer immune responses during the process called immunogenic cell death (ICD) (Fucikova et al., 2020; Rapoport et al., 2020). Beyond the controversy of HMGB1, the roles of HMGB2/3 in cancers are unclear, especially in the context of the tumor immune microenvironment (TIME).

In this work, we comprehensively analyzed the expression, genetic alternations, clinicopathological and prognostic relevance, and underlying mechanisms of HMGBs in pan-cancer. Since biomarkers reflecting TIME, including tumor immune-infiltrating cells (TIICs) and ICP gene expression, and tumor intrinsic features, including microsatellite instability (MSI) and tumor mutational burden (TMB), may predict immunotherapy efficacy (Duffy and Crown, 2019), correlations between HMGB expression and these factors were investigated. This study may offer novel insights into HMGBs’ potential values in cancer immunotherapy.

## Materials and Methods

### Analysis of High Mobility Group Box Genes Expression in Cancers

The differential mRNA expression of HMGBs between human cancers and paired normal controls was analyzed using Oncomine (https://www.oncomine.org) (Rhodes et al., 2007) and Gene Expression Profiling Interactive Analysis 2 (GEPIA2) (http://gepia2.cancer-pku.cn/) (Tang et al., 2017). In the GEPIA2 portal, the data of 33 types of cancers were from the Cancer Genome Atlas (TCGA), and the normal data were combined TCGA and Genotype Tissue Expression (GTEx). The screening criteria were limited to |fold change (FC)| > 2 and a *p* value <0.01 for both portals.

### Analysis of the Prognostic Value of High Mobility Group Box Genes in Cancers

Associations between HMGB expression and overall survival (OS) and relapse-free survival (RFS) of patients with diverse TCGA cancers were evaluated by five databases, Kaplan–Meier (KM) Plotter (http://www.kmplot.com/) (Nagy et al., 2021), Long-term Outcome and Gene Expression Profiling Database of pan-cancers (LOGpc, http://bioinfo.henu.edu.cn/DatabaseList.jsp), SurvExpress (http://bioinformatica.mty.itesm.mx:8080/Biomatec/SurvivaX.jsp) (Aguirre-Gamboa et al., 2013), Tumor IMmune Estimation Resource (TIMER) (http://timer.cistrome.org) (Li Z. et al., 2020), and GEPIA2. Here, patients were divided into high- and low-expression groups by median.

### Analysis of the Clinicopathological Relevance of High Mobility Group Box Genes in Cancers

Associations between HMGB expression and clinicopathological features, including major stages and tumor grades of patients with diverse cancers, were explored using TCGA data by UALCAN (http://ualcan.path.uab.edu) (Chandrashekar et al., 2017).

### Identification of Genetic Alternations of High Mobility Group Box Genes in Cancers

Genetic alternations of HMGBs including mutations, structural variants, and copy number alterations were analyzed by cBioPortal (http://www.cbioportal.org) (Cerami et al., 2012; Gao et al., 2013), using the “TCGA PanCancer Atlas” datasets. Associations between the global alternation of HMGBs and patient’s survivals in pan-cancer were also analyzed; here, samples were split into “altered” and “unaltered” groups.

### Analysis of Correlations Between High Mobility Group Box Genes Expression and Immune Infiltrates and Immune Checkpoint Genes in Cancers

Correlations between HMGB expression and the infiltration of diverse TIICs, including CD8 T cells, CD4 T cells, helper T (Th) 1 cells, Th2 cells, regulatory T cells (Tregs), natural killer (NK) cells, global macrophages, M1/M2 macrophages, neutrophils, myeloid dendritic cells (mDCs), B cells, and myeloid-derived suppressor cells (MDSCs), were explored using the TIMER portal. Correlations between HMGB expression and the infiltration of Th17 were assessed using TISIDB (http://cis.hku.hk/TISIDB) (Ru et al., 2019). Forty-three ICP genes were selected incorporating three review articles (Marin-Acevedo et al., 2021b) (Marin-Acevedo et al., 2021a) (Marin-Acevedo et al., 2018); correlations between the expression of HMGBs and these ICP genes were analyzed using TIMER.

### Analysis of Correlations Between High Mobility Group Box Genes Expression and Microsatellite Instability and Tumor Mutational Burden in Cancers

The RNA sequence data of 33 kinds of TCGA cancers were downloaded from the Genomic Data Commons (GDC) portal (https://portal.gdc.cancer.gov/). MSI (Bonneville et al., 2017) and TMB (Thorsson et al., 2019) data were derived from two previous studies, respectively. Correlations between HMGB expression and MSI and TMB were analyzed using R software version 4.0.3.

### Gene Set Enrichment Analysis for the Correlated Genes of High Mobility Group Box

Correlated genes of *HMGB1* in ACC (*n* = 79), *HMGB2* in LGG (*n* = 516), and *HMGB3* in BRAC (*n* = 1093) were explored using the LinkFinder module of the LinkedOmics platform (Vasaikar et al., 2018). Then, the significantly correlated genes of the *HMGB1/2/3* were respectively sequenced to perform gene set enrichment analysis (GSEA), using Web-based Gene SeT Analysis Toolkit (WebGestalt) (Liao et al., 2019). GSEA was conducted for gene ontology (GO) and Kyoto Encyclopedia of Genes and Genomes (KEGG) pathway categories. GO categories included biological process (BP), cellular component (CC), and molecular function (MF) aspects. The category size was restricted between 5 and 2,000, and the number of permutations was limited up to 1,000. A gene set with a false discovery rate (FDR) < 0.05 was considered significantly enriched.

### Statistical Analysis

A comparison of the mRNA expression was performed using Student’s t-test (Oncomine and UALCAN) or one-way ANOVA test (GEPIA2). Survival curves were plotted using the Kaplan–Meier method, and the log-rank test was performed to identify differences and calculate *p* values. Associations between gene expression and survival were estimated using Cox proportional regression to generate the hazard ratio (HR) and 95% confidence interval (CI). Spearman’s method was applied to analyze correlations between gene expression and the infiltration level of TIICs, MSI, and TMB. Correlations between any two genes were evaluated using the Pearson test. Correlation strength was measured by correlation coefficient (*r)* values: 0.00–0.39, 0.40–0.59, and 0.60–1.0 were weak, moderate, and strong, respectively. All tests were two-tailed paired, and *p* values <0.05 were considered statistically significant.

## Results

### Expression of High Mobility Group Box Genes in Cancers

**FIGURE 1:**
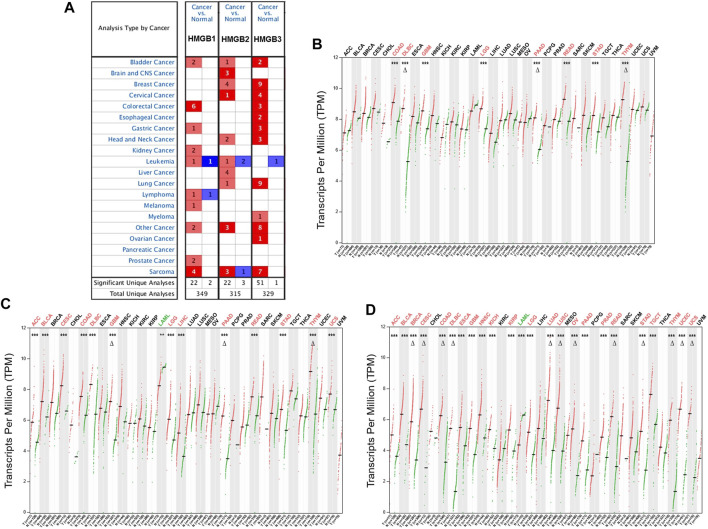
The differential expression of High Mobility Group Box (HMGBs) between cancers and normal controls. **(A)** A summary of the datasets in which HMGBs were significantly up- (red) or down- (blue) expressed in cancers, compared with normal controls (Oncomine). Numbers in cells represent dataset counts. The expression of **(B)** HMGB1, **(C)** HMGB2, and **(D)** HMGB3 in TCGA cancers and paired normal controls (GEPIA2). A black font indicates no significant difference; red or green fonts indicate significant up- or down-expression, respectively, with |fold change (FC)| > 2 and *p* values <0.01. ^***^ *p* < 0.001; ^Δ^|FC| > 4 and *p* < 0.01.

Initially, the results from the Oncomine database showed that HMGB1/2/3 were significantly highly expressed in a total of 22, 22, and 51 datasets, whereas they were lowly expressed in two, three, and one datasets of various cancers, respectively, compared with paired normal controls (Figure 1A). Except for several datasets of leukemia, lymphoma, and sarcoma, HMGBs were consistently up-expressed in most human cancers.

In the GEPIA2 database, *HMGB1/2/3* were significantly differentially expressed in a total of 8, 14, and 24 types of TCGA cancers, respectively, compared with the corresponding normal controls (Figures 1B–D). In detail, *HMGB1/2/3* was uniformly up-expressed in eight kinds of cancers, including colon adenocarcinoma (COAD), lymphoid neoplasm diffuse large B-cell lymphoma (DLBC), glioblastoma multiforme (GBM), brain lower grade glioma (LGG), pancreatic adenocarcinoma (PAAD), rectum adenocarcinoma (READ), stomach adenocarcinoma (STAD), and thymoma (THYM). *HMGB2/3* was highly expressed in four kinds of cancers, including adrenocortical carcinoma (ACC), bladder urothelial carcinoma (BLCA), cervical squamous cell carcinoma and endocervical adenocarcinoma (CESC), and uterine carcinosarcoma (UCS), while they were down-expressed in acute myeloid leukemia (LAML). Besides, *HMGB2* was upregulated in liver hepatocellular carcinoma (LIHC). *HMGB3* was upregulated in 11 other types of cancers, including breast invasive carcinoma (BRCA), esophageal carcinoma (ESCA), head and neck squamous cell carcinoma (HNSC), kidney chromophobe (KICH), kidney renal clear cell carcinoma (KIRC), lung adenocarcinoma (LUAD), lung squamous cell carcinoma (LUSC), ovarian serous cystadenocarcinoma (OV), prostate adenocarcinoma (PRAD), testicular germ cell tumors (TGCT), and uterine corpus endometrial carcinoma (UCEC). To be short, HMGB expression was significantly elevated in most cancers, except that *HMGB2/3* were downregulated in LAML. Moreover, *HMGB3* was the most universally overexpressed among the HMGB family.

### Prognostic Significance of High Mobility Group Box Genes in Cancers

**FIGURE 2:**
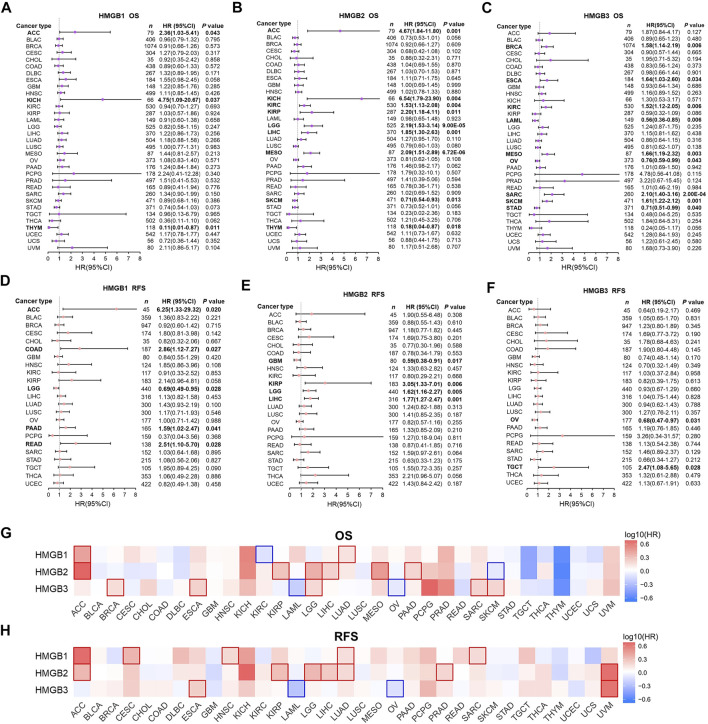
Prognostic significance of High Mobility Group Box (HMGBs) in cancers. Associations between HMGB expression and **(A–C)** OS and **(D–F)** RFS of patients with various cancers. Heat maps showing relations of HMGB expression with **(G)** OS and **(H)** RFS of various cancers (GEPIA2). Results with significance are framed; red or blue frames indicate high or low survival risk, respectively. OS, overall survival; RFS, relapse-free survival; HR, hazard ratio; CI, confidence interval.

In the first step, associations between HMGB expression and OS and RFS of patients with diverse cancers were evaluated integrating LOGpc, KM Plotter, SurvExpres, and TIMER platforms (Supplementary Table S1). We found that a higher expression of *HMGB1* was significantly related with worse OS of patients with ACC (HR = 2.36, *p* = 0.043) and KICH (HR = 4.75 *p* = 0.037), whereas a better OS of patients with THYM was found (HR = 0.11, *p* = 0.011) (Figure 2A). An elevated expression of *HMGB2* was significantly linked to shorter OS of patients with ACC (HR = 4.67, *p* = 0.001), KICH (HR = 6.54, *p* = 0.004), KIRC (HR = 1.53, *p* = 0.004), KIRP (HR = 2.20, *p* = 0.011), LGG (HR = 2.19, *p* = 9.00E-05), LIHC (HR = 1.85, *p* = 0.001), and MESO (HR = 2.09, *p* = 6.72E-06), and a longer OS of patients with SKCM (HR = 0.71, *p* = 0.013) and THYM (HR = 0.18, *p* = 0.018) (Figure 2B). *HMGB3* overexpression implied unfavorable OS of patients with BRCA (HR = 1.58, *p* = 0.006), ESCA (HR = 1.64, *p* = 0.034), KIRC (HR = 1.52, *p* = 0.006), MESO (HR = 1.66, *p* = 0.003), SARC (HR = 2.10, *p* = 2.00E-04), and SKCM (HR = 1.61, *p* = 0.001), but better OS of patients with LAML (HR = 0.56, *p* = 0.006), OV (HR = 0.76, *p* = 0.043), and STAD (HR = 0.71, *p* = 0.040) (Figure 2C).

Apart from several cancer types with insufficient sample size that were not analyzed, we further found that *HMGB1* upregulation was significantly linked with unfavorable RFS of ACC, COAD, PAAD, and READ, but better RFS of LGG (Figure 2D). *HMGB2* high expression suggested worse RFS of KIRP, LGG, LIHC, but better RFS of GBM (Figure 2E). *HMGB3* up-expression implied better RFS of OV but worse RFS of TGCT (Figure 2F).

Second step, heat maps exhibiting HMGBs’ prognostic values were generated by GEPIA (Figures 2G,H). Here, *HMGB1* high expression indicated both worse OS and RFS of ACC and LUAD; worse RFS of CESC, HNSC, and SARC; and better OS of KIRC. *HMGB2* up-expression suggested both worse OS and RFS of ACC, KIRP, LGG, and LIHC; worse OS of MESO and PAAD; worse RFS of LUAD and PRAD; and better OS of SKCM. *HMGB3* upregulation signified both poorer OS and RFS of ESCA; worse OS of BRCA, LGG, SARC, and SKCM; and better OS and RFS of LAML and OV. We took the intersection of the findings of the two steps of survival analyses to improve the robustness, which was provided in the discussion section.

### Clinicopathological Relevance of High Mobility Group Box Genes in Cancers

**FIGURE 3:**
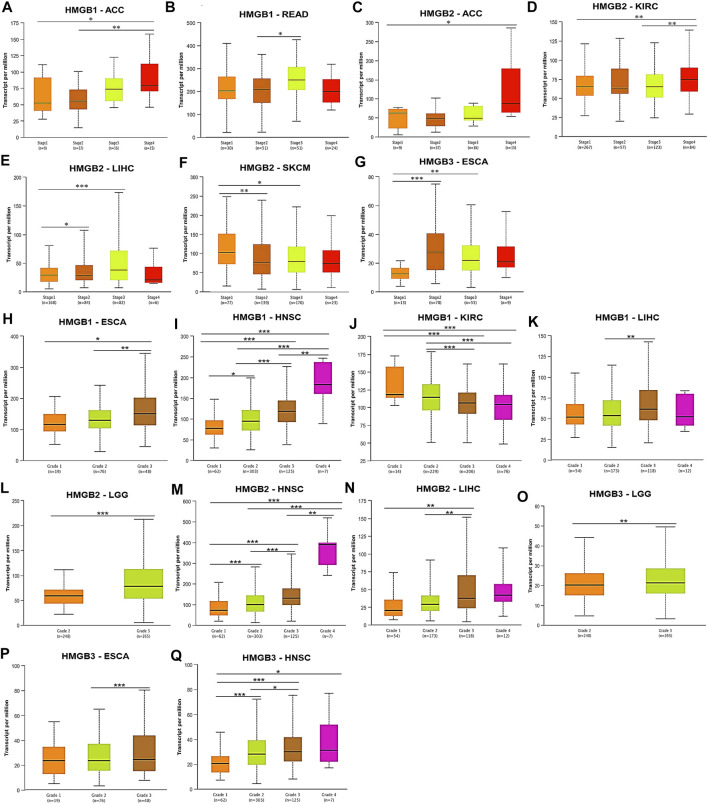
Clinicopathological relevance of High Mobility Group Box (HMGBs) in cancers. Associations of the expression of **(A–B)** HMGB1, **(C–F)** HMGB2, and **(G)** HMGB3 with pathological stages of several cancers. Associations of the expression of **(H–K)** HMGB1, **(L–N)** HMGB2, and **(O–Q)** HMGB3 with tumor grades of several cancers (UALCAN). ^*^ *p* < 0.05, ^**^ *p* < 0.01, ^***^ *p* < 0.001.

Subsequently, correlations between HMGB expression and clinicopathological characteristics of diverse cancers were investigated. We found that *HMGB1* expression was elevated with the stage progression of ACC and READ. *HMGB1* expression was significantly higher in Stage-IV ACC and Stage-III READ than in Stage-I/II ACC and Stage-II READ, respectively (*p* < 0.05) (Figures 3A,B). *HMGB2* expression was elevated as stages of ACC, KIRC, and LIHC were promoted, while stages of SKCM improved. The expression of *HMGB2* was significantly higher in Stage-IV ACC, Stage-IV KIRC, and Stage-II/III LIHC than in Stage-I ACC, Stage-I/III KIRC, and Stage-I LIHC, respectively. In contrast, *HMGB2* expression was significantly lower in Stage-II/III SKCM, compared to that in Stage-I ones (*p* < 0.05) (Figures 3C–F). *HMGB3* expression was elevated in Stage-II/III ESCA, compared with that in Stage-I ones (*p* < 0.05) (Figure 3G).

What is more, tumor grades of HNSC were significantly increased with the elevation of HMGB expression (Figures 3I,M,Q), while an opposite trend was observed for *HMGB1* expression in KIRC (Figure 3J). Significantly, *HMGB1/2* expression was higher in Grade-3 tumors of LIHC than in Grade-2 (and −1) ones (*p* < 0.01) (Figures 3K,N). *HMGB1/3* expression was higher in Grade-3 tumors of ESCA than in Grade-2 (and −1) ones (*p* < 0.05) (Figures 3H,P). *HMGB2/3* expression was higher in Grade-3 tumors of LGG than in Grade-2 ones (*p* < 0.01) (Figures 3L,O). Collectively, HMGB up-expression indicated the clinicopathological advancement of ACC, ESCA, HNSC, LIHC, LGG, and READ and the alleviation of KIRC and SKCM.

### Genetic Alternations of High Mobility Group Box Genes in Cancers

**FIGURE 4:**
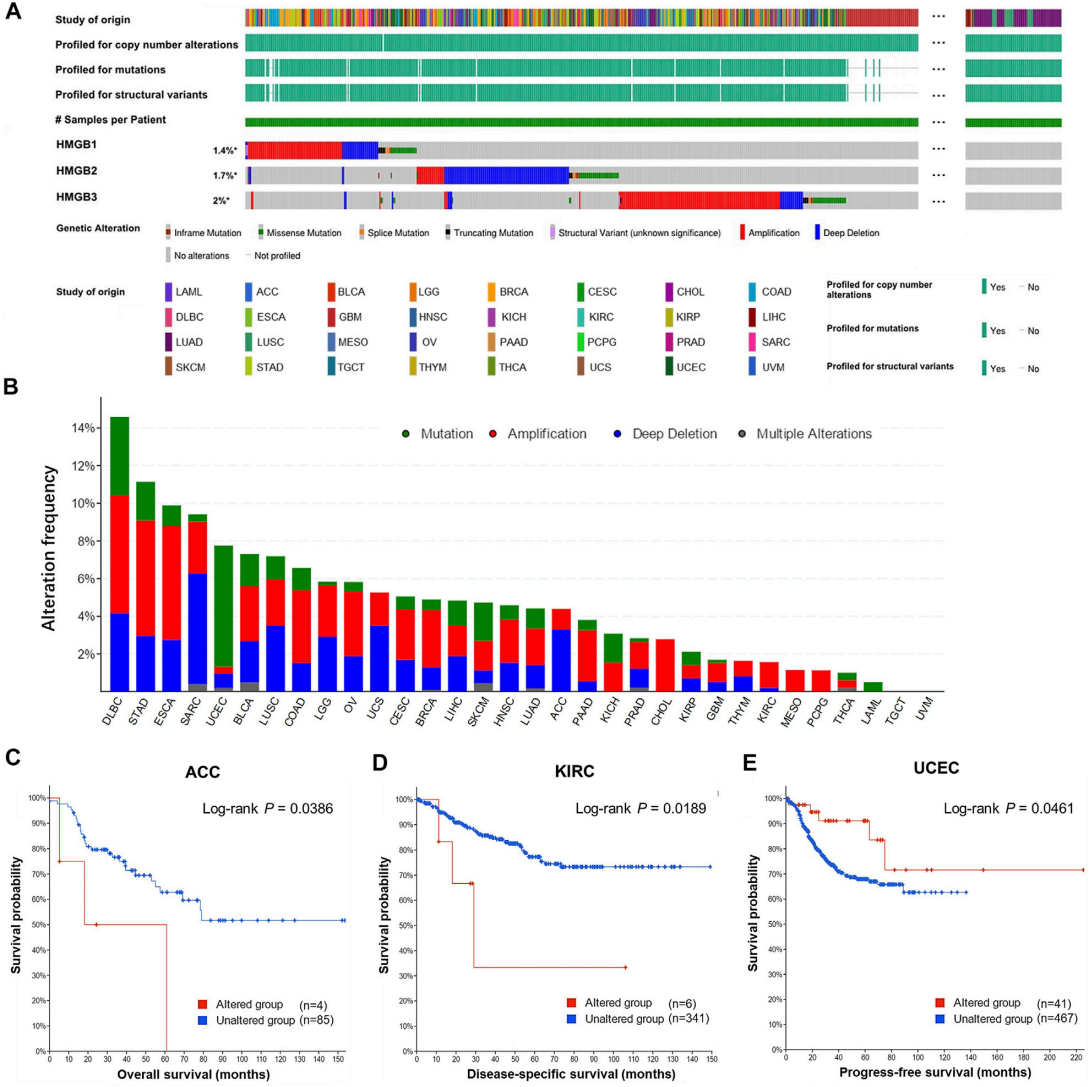
Genetic alternations of High Mobility Group Box (HMGBs) in cancers. **(A)** An overview of the genomic alternations of HMGBs occurred in pan-cancer. **(B)** The alternation frequency of HMGBs in cancers. Associations of HMGBs’ global alternation with the survival of patients with **(C)** ACC, **(D)** KIRC, and **(E)** UCEC (cBioPortal).

Overall, genetic alternations of HMGBs were identified in a total of 529 (4.83%) out of 10,953 samples, including in-frame mutation, missense mutation, splice mutation, truncating mutation, structural variant, amplification, and deep deletion (Figure 4A). Among all the cancers, HMGBs altered the most frequently in DLBL, with an incidence rate of 14.58%, followed by STAD (11.14%) and ESCA (9.89%) (Figure 4B). *HMGB3* was the most frequently altered one within HMGBs (221 out of 10950 samples).

The occurrence of HMGB alternations was significantly related to poorer OS of ACC (Figure 4C) and poorer disease-specific survival of KIRC (Figure 4D), but a better progression-free survival of UCEC (*p* < 0.05) (Figure 4E). Apart from these, no significant survival relevance was found for other cancer types.

### Correlations Between High Mobility Group Box Genes Expression and Immune Infiltrates and Immune Checkpoint Genes in Cancers

**FIGURE 5:**
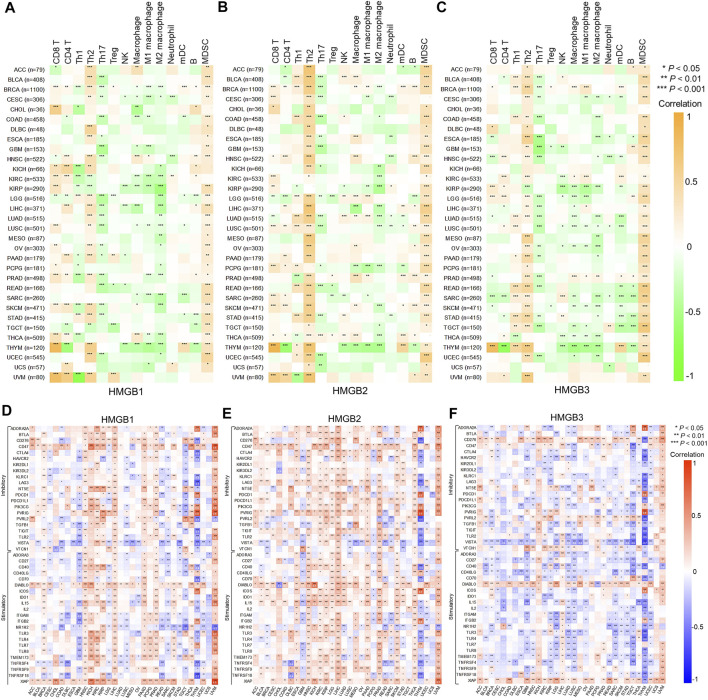
Correlations between High Mobility Group Box (HMGBs) expression and **(A–C)** immune infiltration and **(D–F)** ICP genes expression in cancers. Th, helper T cell; Treg, regulatory T cell; NK cell, natural killer cell; mDC, myeloid dendritic cell; MDSC, myeloid-derived suppressor cell.

Correlations between HMGB expression and infiltration levels of TIICs were investigated integrating TIMER and TISID. Generally, HMGBs were significantly positively correlated with the infiltration of Th2 cells and MDSCs and negatively correlated with that of Th17 cells in pan-cancer (Figures 5A–C). Specifically, *HMGB1* expression showed positive correlations with the infiltration of CD8 and CD4 T cells, but negative ones with that of Th1 cells and macrophages in BRCA, KIRC, KIRP, LIHC, PRAD, SKCM, and THCA. *HMGB2* expression was positively or negatively correlated with the infiltration of diverse TIICs in BRCA, LGG, LUAD, PCPG, and THCA, without a consistent pattern. *HMGB3* expression exhibited negative correlations with the infiltration of macrophage lineages in KIRP, LGG, LUAD, LUSC, OV, SARC, SKCM, TGCT, and THCA. Notably, strong to very strong correlations were observed as follows: HMGB expression and the infiltration of CD8 T cells and (or) Th2 cells in THYM and UVM; *HMGB2* expression and Th2 cell infiltration in ACC, BLCA, LIHC, and MESO; and *HMGB2/3* expression and MDSC infiltration in UCEC.

Inhibitory and stimulatory ICPs regulate immune escape and immune efficacy, respectively. Here, we explored correlations between the expression of HMGBs and 43 ICP genes (21 inhibitory and 22 stimulatory). In general, the relationships between the expression of HMGBs and inhibitory or stimulatory ICP genes were isotropic. To highlight, strong correlations were identified for HMGB expression with many ICP genes of THYM, with mostly negative relations, as well as *HMGB1/2* expression with numerous ICP genes of UVM. Besides, significant positive correlations between the expression of HMGBs and ICP genes were found in the following cancers: *HMGB1* in HNSC, LIHC, PAAD, and PRAD and *HMGB2* in HNSC, KIRC, KIRP, LGG, LIHC, PRAD, THCA, and SKCM. In contrast, significant negative correlations were found as follows: HMGBs in GBM and *HMGB3* in KIRP, LGG, LUAD, LUSC, and TGCT. (Figures 5D–F).

### Correlations Between High Mobility Group Box Genes Expression and Microsatellite instability and Tumor Mutational Burden in Cancers

**FIGURE 6:**
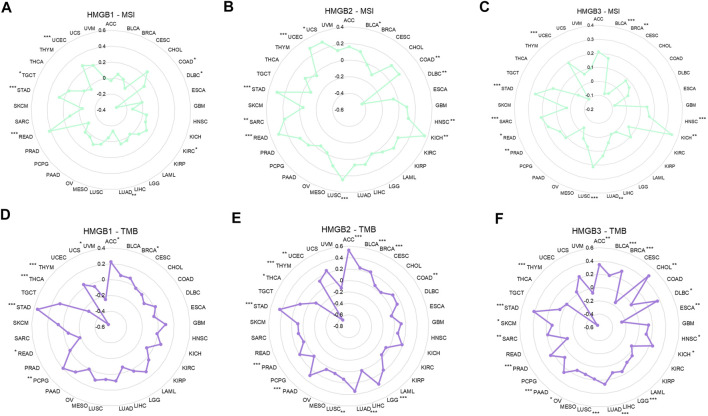
Correlations between High Mobility Group Box (HMGBs) expression and the **(A–C)** MSI and **(D–F)** TMB of cancers. MSI, microsatellite instability; TMB, tumor mutational burden.

Among 33 kinds of cancers, *HMGB1/2/3* expression was significantly positively correlated with the MSI of 6 (18.2%), 10 (30.3%), and 10 (30.3%) types of cancers but negatively correlated with the MSI of 2 (6.0%), 1 (3.0%), and 1 (3.0%) types of cancers respectively (Figures 6A–C).

As for the TMB, *HMGB1/2/3* expression was significantly positively correlated with the TMB of 4 (12.1%), 11 (33.3%), and 16 (48.5%) kinds of cancers but negatively correlated with the TMB of 4 (12.1%), 2 (6.0%), and 2 (6.0%) kinds of cancers, respectively (Figures 6D–F). Particularly, HMGB expression had almost strong negative correlations with the TMB of THYM. In addition, positive relationships with both MSI and TMB were identified for *HMGB1* in STAD; *HMGB2* in STAD, BLCA, UCEC, LUSC, and COAD; and *HMGB3* in STAD, LUAD, PRAD, LUSC, BLCA, SAR, HNSC, and KICH.

### Potential Functions of the Correlated Genes of High Mobility Group Box Genes

**FIGURE 7:**
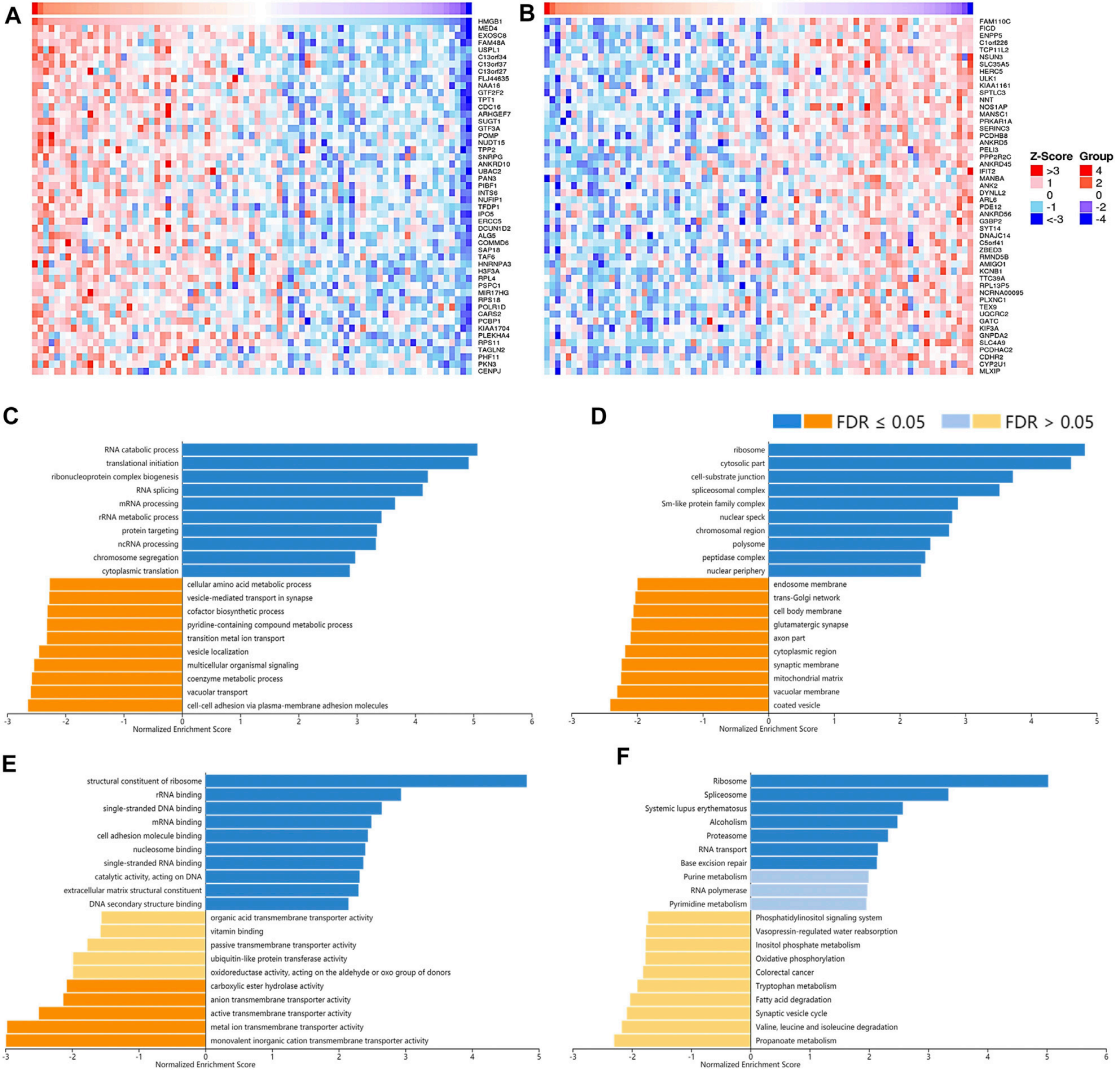
The corrected genes of High Mobility Group Box 1 (HMGB1) in ACC and GSEA results. The top 50 genes significantly **(A)** positively and **(B)** negatively correlated with HMGB1 in ACC (LinkedOmics). The top 20 significantly enriched **(C)** GO-BP, **(D)** GO-CC, **(E)** GO-MF, and **(F)** KEGG pathway terms of HMGB1 correlated genes based on GSEA. A bar represents a normalized enrichment score for a term, which in orange or blue represents negatively or positively enriched, respectively.

To understand the potential mechanisms behind the differential expression and immunological relevance of HMGBs in different cancer types, we explored correlated genes of *HMGB1/2/3* in three representative cancer types and performed GSEA for them, respectively. A total of 3,452 genes were found significantly correlated with *HMGB1* in ACC, and the top 50 of the positively and negatively correlated ones are shown in Figures 7A,B respectively. The results of GSEA illuminated that the positively correlated genes of *HMGB1* in ACC might comprise the ribosome, cytosolic part, cell–substrate junction, etc., and partake in RNA metabolic processes and translation. Signaling pathways of the ribosome, spliceosome, and purine metabolism were involved. Nevertheless, the negatively correlated genes of *HMGB1* might comprise coated vesicles and vacuolar membranes and be involved in cell–cell adhesion *via* plasma–membrane adhesion molecules and various transmembrane transports (Figures 7C–F).

**FIGURE 8:**
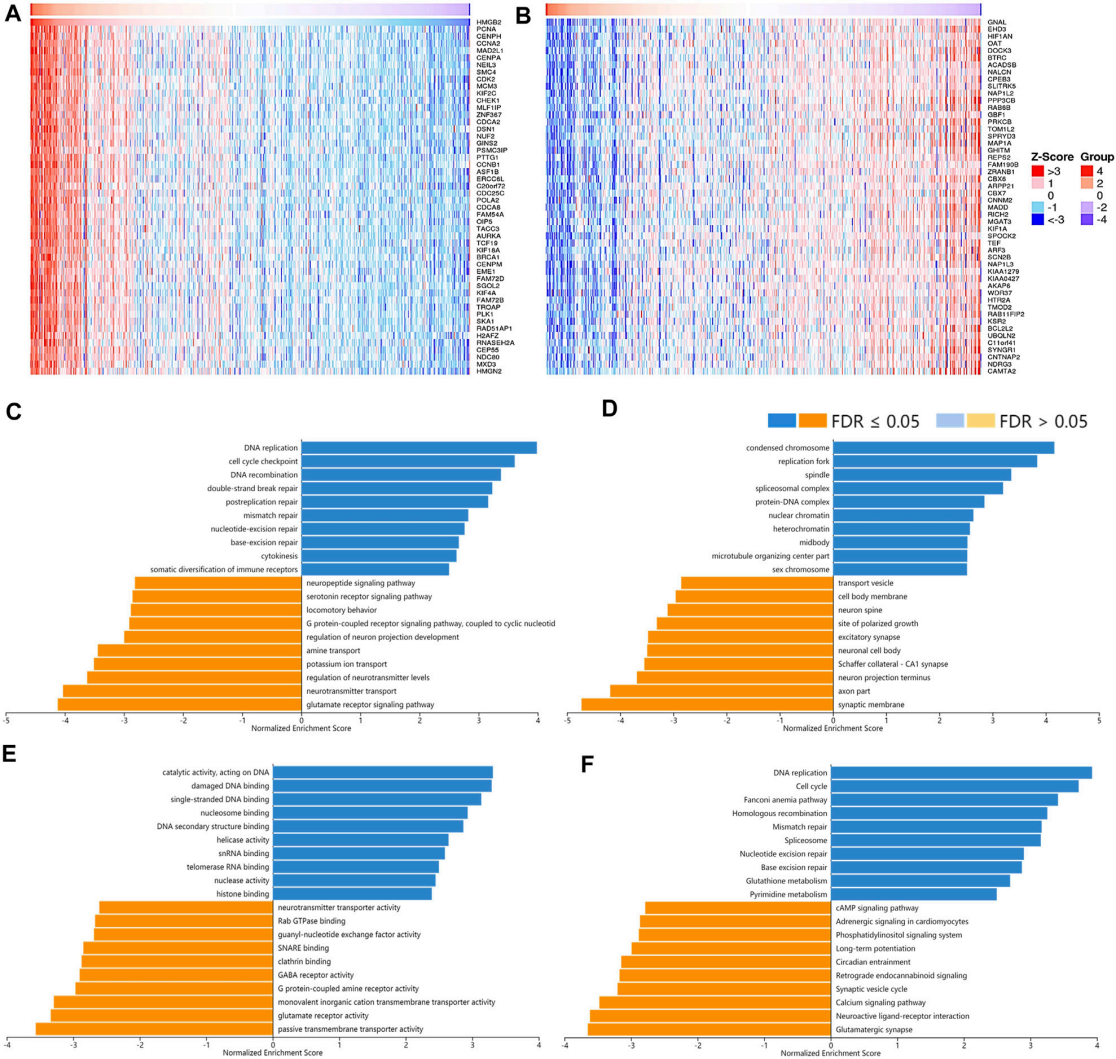
The correlated genes of High Mobility Group Box 2 (HMGB2) in LGG and GSEA results. The top 50 genes significantly **(A)** positively and **(B)** negatively correlated with HMGB2 in LGG (LinkedOmics). The top 20 significantly enriched **(C)** GO-BP, **(D)** GO-CC, **(E)** GO-MF, and **(F)** KEGG pathway terms of HMGB2 correlated genes based on GSEA. A bar represents a normalized enrichment score for a term, which in orange or blue represents negatively or positively enriched respectively.

A total of 12,967 genes were found significantly correlated with *HMGB2* in LGG (Figures 8A,B). The positively correlated genes of *HMGB2* in LGG might be components of the chromosome, replication fork, and spindle and be responsible for BPs and pathways regulating cell cycle checkpoint, DNA replication, recombination, and damage repair, as well as somatic diversification immune receptors. In contrast, the negatively correlated genes of *HMGB2* might consist of the synaptic membrane, axon part, neuron projection terminus, and transport vesicles and be involved in signaling pathways of glutamate receptor, neurotransmitter transport, G protein-coupled receptor, and cAMP (Figures 8C–F).

**FIGURE 9:**
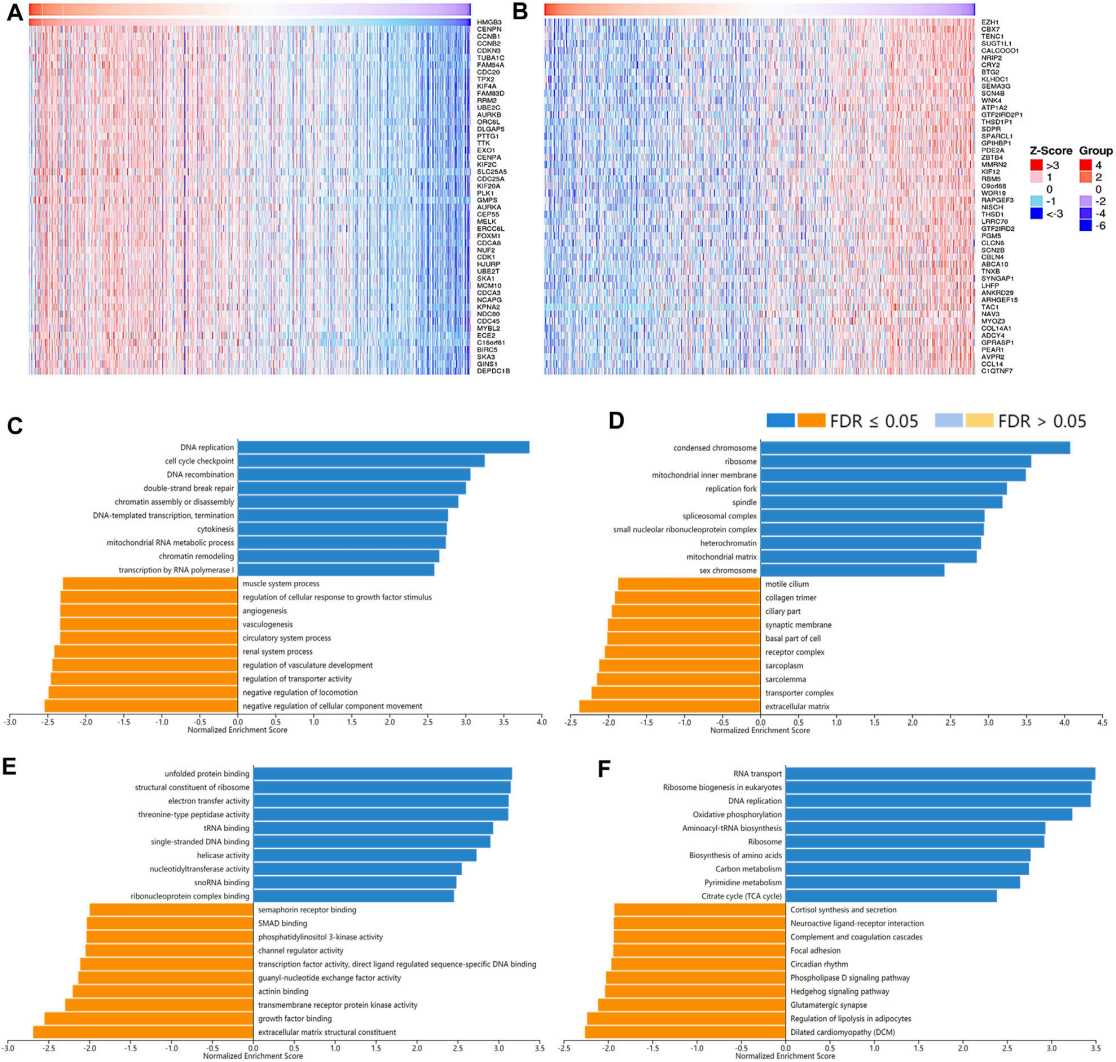
The correlated genes of High Mobility Group Box 3 (HMGB3) in BRAC and GSEA results. The top 50 genes significantly **(A)** positively and **(B)** negatively correlated with HMGB3 in BRAC (LinkedOmics). The top 20 significantly enriched **(C)** GO-BP, **(D)** GO-CC, **(E)** GO-MF, and **(F)** KEGG pathway terms of HMGB3 correlated genes based on GSEA. A bar represents a normalized enrichment score for a term, which in orange or blue represents negatively or positively enriched, respectively.

As for the correlated genes of *HMGB3* in BRAC, 14,028 genes were significantly observed in all (Figures 9A,B). Similarly, the positively correlated genes of *HMGB3* in BRAC were generally chromosome structures and partake in BPs and pathways related to replication, DNA repair, and chromatin remodeling. Additionally, pathways of amino acid biosynthesis, carbon metabolism, and citrate cycle were also enriched. The negatively correlated genes of *HMGB3* might consist of extracellular matrix and transporter complexes, which contributed to angiogenesis and the negative regulation of locomotion. Moreover, the signaling pathways of Hedgehog and focal adhesion were related (Figures 9C–F).

## Discussion

This study extracted potential values of HMGBs in various cancers, especially in the context of immunotherapy.

From the outset, we found that HMGBs were significantly up-expressed in various TCGA cancers, except that *HMGB2/3* were down-expressed in LAML. Despite that the overexpression of *HMGB1* in cancers was the most prevalently reported (Niu et al., 2020), we found that *HMGB3* was highly expressed in the largest variety of cancers and altered most frequently. Integrating the results of two steps of survival analyses, high expression of HMGBs suggested unfavorable prognosis in the following cancers: *HMGB1* in ACC; *HMGB2* in ACC, KIRP, LGG, LIHC, MESO; and *HMGB3* in BRCA, ESCA, SARC, and SKCM. In contrast, favorable prognostic indications were found for the up-expression of *HMGB2* in SKCM, as well as *HMGB3* in OV and LAML. By the way, the global alternation of HMGBs was linked with worse outcomes of ACC and KIRC, but a better outcome of UCEC. In addition, elevated HMGB expression indicated clinicopathological advances in these cancers: *HMGB1* in ACC, HNSC, and ESCA; *HMGB2* in ACC, HNSC, KIRC, LGG, and LIHC; and *HMGB3* in ESCA and HNSC. Conversely, the up-expression of *HMGB1* and *HMGB2* suggested clinicopathological alleviation of KIRC and SKCM, respectively.

The findings of some earlier studies were consistent with ours. Nguyen *et al.* reported that HMGB1 was related to the clinical and pathological characteristics of HNSC (Nguyen et al., 2016). Kwon *et al.* stated that HMGB2 overexpression implied the aggressiveness and worse prognosis of LIHC (Kwon et al., 2010). In an experimental study, HMGB2 was observed to be highly expressed in melanoma, whose silence impeded cell proliferation and invasion, yet promoted cell cycle arrest and apoptosis, leading to melanoma regression, indicating that HMGB2 contributed to melanoma promotion (Mo et al., 2019). As for HMGB3, several experiments revealed that it was upregulated by diverse noncoding RNAs, which in turn fomented malignant behaviors and even immune escape of breast cancer cells (Gu et al., 2019; Chen et al., 2021; Yu et al., 2021). A recent study indicated that hypermethylation of the promoter of miR-216a upregulated HMGB3, which then promoted ESCA (Sun et al., 2021). Paradoxically, HMGB3 high expression was shown to facilitate cisplatin resistance of ovarian cancer cells; however, we found it a favorable prognostic indicator of OV (Mukherjee et al., 2019).

It is well known that CD8 T cells, NK cells, and Th1 cells exert anticancer immunity, while TAMs, MDSCs, Tregs, Th2 cells, and tolerogenic mDCs foster pro-cancer immune escape in the TIME (Zhang and Zhang, 2020; Saillard et al., 2021). ICIs can unleash preexisting tumor-infiltrating lymphocytes (TILs) and restore their lethality to cancer cells. Increased density of TILs, particularly CD8 T cells, improved the therapeutic responses and outcomes of patients across various malignancies (Nishino et al., 2017). However, only patients with high ICP expression may benefit from ICI therapy; a most adopted predictor is PD-L1 expression (Randrian et al., 2021). In this study, we investigated correlations between HMGB expression and both immune infiltration and ICP gene expression, to learn their involvements in the TIME and predictive capacities for the response to ICI therapy. Generally, HMGBs were positively or negatively associated with both immune-stimulative TIICs/ICP genes and immunosuppressive TIICs/ICP genes in pan-cancer, suggesting that they might modulate the TIME in both provocative and inhibitory ways. However, we inferred that HMGBs were inclined to induce overall immunosuppression in the TIME, since we found that they had uniformly positive correlations with the infiltration of Th2 cells and MDSCs in pan-cancer. Indeed, interactions between HMGB1 and its receptors are critical for the differentiation and activation of MDSCs (Jin et al., 2020) and Tregs (Wild et al., 2012) and the upregulation PD-L1 in the TIME (Wang et al., 2019). Besides, it was evident that HMGB1 could induce a dominance of Th2-type response in inflammation (Ma et al., 2015).

HMGB expression showed strong correlations with TIICs and ICP genes in THYM and UVM, signifying their outstanding positions in the TIME of the two kinds of cancers. For HNSC, HMGB up-expression suggested increased ICP gene expression and rising density of immune-suppressive Th2 cells, macrophages, and MDSCs, which might contribute to the disease progress. *HMGB2* up-expression indicated elevated infiltration of Th2 cells and MDSCs and (or) ICP gene expression in ACC, KIRP, LGG, LIHC, and MESO, with medium to strong correlation strength, which might partly explain the poor survival of patients with these cancers. Oppositely, *HMGB2* upregulation might benefit SKCM patients through activating CD8 T cells and stimulatory ICPs. In fact, an earlier study indicated that HMGB2 participated in the cytoplasmic chromatin recognition and the subsequent response to anticancer ICP blockade (Zhao et al., 2020). A high *HMGB3* expression was a detrimental prognostic factor for BRCA, ESCA, SARC, and SKCM, which might blame on its negative relationships with various stimulatory ICPs and the infiltration of CD8 T cells but positive interactions with Th2 cells and MDSCs. In contrast, the beneficial role of *HMGB3* in OV might partially be explained by the scarce immunological interactions. All the above manifested HMGBs might partake in the development of these cancers through coordinating TIICs and ICPs, thus potentially serving as immunotherapy targets. Seeing from another angle, HMGBs could also be used as predictive biomarkers for immunotherapeutic response in some cancers. This is because, for a cancer type in which the expression of ICPs and HMGBs was positively correlated, a high HMGB expression might predict a better response to ICI therapy.

Microsatellites are short DNA stretches tandemly repeated throughout the genome, and MSI occurs when the genome gains or loses ≥ one repeat(s). TMB represents the total number of mutations per DNA megabase (Duffy and Crown, 2019). High MSI is an underlying process contributing to high TMB, and higher MSI or TMB levels may generate potent neoantigens for recognition by immune surveillance, thus increasing immunotherapy responses (Duffy and Crown, 2019; Veigas et al., 2021). We found that HMGBs were significantly positively correlated with MSI and (or) TMB in diverse cancers, suggesting that high HMGB expression might predict clinical benefits from immunotherapy for patients with these cancers. Within the HMGB family, *HMGB3* expression was associated with MSI and (or) TMB in most cancer types, consistent with its highest alternation occurrence rate in pan-cancer. Integrating the significance of prognosis, TIICs, ICP genes, and MSI and (or) TMB, we induced that HMGBs might be promising immunological targets for the following cancers: *HMGB1* for ACC and KIRC; *HMGB2* for ACC and LGG; and *HMGB3* for BRAC, SARC, SKCM, and OV.

Genes positively correlated with HMGBs might be their potential co-expressed genes, which were similarly chromosome components regulating DNA replication, transcription, damage repair, chromatin remodeling, and cell cycle. These functions of HMGBs favor cancer cells to maintain their nature of continuous proliferation and protect them from therapy-caused DNA damages (Cámara-Quílez et al., 2020). What is more, genes positively correlated with HMGB3 in BRAC were also enriched in pathways of amino acids and carbon metabolism, indicating their participation in cancer metabolic alternations. Beyond intracellular functions, HMGBs, especially HMGB1, can be actively secreted by cancer cells *per s*, infiltrating immune cells, and stromal cells, or passively released from necrotic cells into extracellular milieu in response to various stimuli. Upon HMGBs binding to cell-surface receptors or immune receptors, e.g., receptor for advanced glycation end product (RAGE) and toll-like receptors (TLRs), inflammatory and immune responses are amplified *via* a positive feedback loop (Kang et al., 2013; Musumeci et al., 2014). The durable chronic inflammation then activates multiple downstream pathways, e.g., nuclear factor κB (NF-κB), mitogen-activated protein kinase (MAPK), and phosphatidylinositol 3-kinase (PI3K), to promote cancer through modulating apoptosis, autophagy, invasion, metastasis, and angiogenesis (Bianchi et al., 2017; Lee et al., 2019; Mukherjee and Vasquez, 2020). High levels of HMGB1 can recruit MDSCs, macrophages, neutrophils, immature DCs, and Tregs and increase their T cell inhibitory properties to establish a highly immunosuppressive TIME conducive to immune escape (Gorgulho et al., 2019). Furthermore, HMGB1 interacts with immunomodulatory molecules to hinder immune activities, e.g., T-cell immunoglobulin and mucin domain-containing-3 (TIM-3) (Kwak et al., 2020). Paradoxically, HMGB1 also stimulates TILs and produce anticancer immunity as an immunogenic signal during ICD, which is a kind of cell death caused by chemo- or radiotherapies (Apetoh et al., 2007). To summarize, HMGB up-expression is essential for cancer cells to maintain the hallmarks of unlimited proliferation and permanent inflammation, which made them forceful biomarkers of pan-cancer. HMGBs are intertwined in extensive signaling pathways of inflammation and immunity, thus affecting the immune infiltration and ICP expression in the TIME of cancers. Differences between diverse cancer types might attribute to not only the inherent heterogeneity of cancers but also the inflammation level, cytokines, chemokines, inner receptors, targeted cells, and redox states of HMGBs in the tumor sites (Kang et al., 2013). Despite that knowledge about HMGB2/3 is very limited, they might have similar regulatory patterns with HMGB1 based on their high identities. That said, there is still a long way to go to clarify the specific mechanisms.

## Conclusion

This study observed that HMGBs were significantly differentially expressed in a wide range of cancers. HMGB expression was associated with the prognosis and clinicopathologic characteristics of various cancers, which might be partially explained by their extensive interactions with TIICs and ICPs in the TIME. Besides, HMGB expression was related to MSI and TMB in multiple cancers, which further displayed their potentials as cancer immunotherapy targets and biomarkers for immunotherapeutic response prediction. Therefore, it is necessary to conduct in-depth studies on the immune-related functions of HMGBs, especially HMGB2/3. Besides, we underscored the importance of HMGB1 in ACC and KIRC; HMGB2 in ACC and LGG; and HMGB3 in BRAC, SARC, SKCM, and OV. Although careful validations were warranted, our study might deepen the understanding of the roles of HMGBs in pan-cancer and provide novel insights for future immunotherapy strategies.

## Data Availability

The datasets presented in this study can be found in online repositories. The names of the repositories and accession numbers can be found in the article/Supplementary Material.
